# The relationship between cognitive screeners and everyday functioning in amyloid‐positive participants from the Amsterdam Dementia Cohort

**DOI:** 10.1002/dad2.70233

**Published:** 2026-01-04

**Authors:** Angela van der Putten‐Toorenburg, Elke Butterbrod, Benjamin D. Schalet, Pieter J. van der Veere, Mukrabe E. Tewolde, Merel C. Postema, Elsmarieke van de Giessen, Charlotte E. Teunissen, Argonde C. van Harten, Wiesje M. van der Flier, Sietske A. M. Sikkes

**Affiliations:** ^1^ Faculty of Behavioural and Movement Sciences Department of Clinical Neuro‐ and Developmental Psychology Vrije Universiteit Amsterdam Amsterdam The Netherlands; ^2^ Alzheimer Center Amsterdam Department of Neurology Vrije Universiteit Amsterdam Amsterdam UMC location VUmc Amsterdam The Netherlands; ^3^ Department of Epidemiology and Data Science Vrije Universiteit Amsterdam Amsterdam UMC Amsterdam The Netherlands; ^4^ Amsterdam Neuroscience‐Neurodegeneration Vrije Universiteit Amsterdam Amsterdam The Netherlands; ^5^ Department of Radiology and Nuclear Medicine Amsterdam Neuroscience Vrije Universiteit Amsterdam Amsterdam UMC Amsterdam The Netherlands; ^6^ Neurochemistry Laboratory Department of Laboratory Medicine Amsterdam Neuroscience Amsterdam UMC Vrije Universiteit Amsterdam Amsterdam The Netherlands

**Keywords:** Alzheimer's disease, clinical meaningfulness, everyday functioning, global cognition, instrumental activities of daily living, preclinical AD

## Abstract

**INTRODUCTION:**

We explored the relationship between cognitive screening outcomes and everyday functioning in Alzheimer's disease (AD).

**METHODS:**

A total of 1228 amyloid‐positive participants were included from the Amsterdam Dementia Cohort. Multiple linear regression analyses assessed the relationship between Mini‐Mental State Examination (MMSE), Montreal Cognitive Assessment (MoCA), and everyday functioning (Amsterdam Instrumental Activities of Daily Living Questionnaire [A‐IADL‐Q‐30]). To link cognitive screeners to functional impairment, we described difficulties across A‐IADL‐Q‐30 items by MMSE and MoCA quartiles.

**RESULTS:**

Both MMSE (*B* = 0.96, 95% confidence interval [CI]0.87–1.04) and MoCA (*B* = 0.79, 95% CI 0.68–0.89) were associated with A‐IADL‐Q‐30. In the lowest MMSE (0–20) and MoCA (0–16) quartiles, filling in forms (both 96%) and managing the household budget (95%–93%) were mostly affected, whereas working (74%) and using a computer (52%–50%) were primarily affected in the highest quartiles (MMSE 28–30/MoCA 25–30).

**DISCUSSION:**

In amyloid‐positive participants, the association between cognition and daily functioning was moderate, reinforcing the importance of assessing both constructs in disease monitoring.

**Highlights:**

- Cognitive screening tools were moderately associated with daily functioning.
- Difficulties in complex daily tasks were present in the higher cognitive performance quartiles.
- Findings suggest that combining cognition and function is required for disease monitoring.

## 1 BACKGROUND

Alzheimer's disease (AD) is characterized by neuropathologic changes, including amyloid beta (Aβ) plaques and tau neurofibrillary tangles in the brain.^1^ As neuropathological burden increases, symptoms emerge and progress^1^ through clinically‐defined stages such as subjective cognitive decline (SCD), mild cognitive impairment (MCI), and dementia. New disease‐modifying treatments (DMTs) reduce amyloid plaques, which translates into a reduced rate of cognitive decline in mild cognitive impairment (MCI) and mild dementia.^2^, ^3^, ^4^ Clinical trials often measure therapy efficacy using cognitive screeners such as the MMSE (Mini‐Mental State Examination).^5^ However, there is ongoing debate about the clinical meaningfulness of a 25%–30% reduction of cognitive decline as measured by such tools.^6^ A change of this magnitude may not necessarily translate into meaningful differences in everyday functioning, highlighting the uncertainty around the real‐world relevance of cognitive screening tools.

In contrast to cognitive screening measures, a decline in instrumental activities of daily living (IADLs) can be more readily interpreted as meaningful. IADL assessments capture concrete everyday tasks, such as managing the household budget or operating domestic appliances, that directly reflect everyday functioning relevant to both patients with dementia and their caregivers. As such, they offer a more tangible indication of meaningful change in daily life. Although recent prognostic prediction models have shown the ability to predict global cognitive screening scores (MMSE) over time in amyloid‐positive individuals, it is not clear how those screening tools relate to everyday functioning.^7^ Previously, global cognitive performance measured with an extensive neuropsychological test battery did not predict everyday functioning in individuals with dementia, although this was assessed in a mixed sample with unknown amyloid status.^8^ Exploring how activities are affected across different cognitive stages can provide valuable insights into which specific, potentially meaningful activities are among the first to deteriorate and which may be preserved for a longer time.

In this study, we aimed to translate global cognitive screening tools to everyday instrumental functioning in amyloid‐positive individuals in a memory clinic setting. Ideally, we aimed to translate scores from one measure into another using a procedure known as score linking, so that the scores could become interchangeable with a high degree of accuracy.^9^ However, this procedure requires that the constructs underlying each measure are largely the same and that they are highly correlated.^9^, ^10^ Given that our pair of constructs (cognition vs daily functioning) is not the same, we proceeded with a prediction procedure based on multiple regression.^11^, ^12^ We hypothesize that lower performance on global screeners is associated with greater difficulties in IADLs.

## 2 METHODS

### 2.1 Participants

In this cross‐sectional study, we included 1228 amyloid‐positive participants from the Amsterdam Dementia Cohort (ADC), a memory clinic cohort consisting of participants who visited the memory clinic of Alzheimer Center Amsterdam between January 2012 and August 2023. All patients underwent a standardized one‐day diagnostic assessment evaluation including medical history; neurologic, physical, and neuropsychological tests; MRI; and lumbar puncture. For inclusion in our study, participants had to be amyloid positive and have a diagnosis of mild cognitive impairment (MCI) (*n* = 228, 19%) or dementia (*n* = 844, 69%). Individuals in whom no cognitive impairments were established, but who were amyloid positive, were categorized as SCD (*n* = 156, 13%). Additional inclusion criteria were completion of the MMSE or MoCA, and completion of the Amsterdam Instrumental Activities of Daily Living Questionnaire (A‐IADL‐Q‐30) by a study partner (i.e., proxy). Educational attainment was self‐reported by patients or their study partners and classified according to the 7‐point Verhage scale. This systematically categorizes individuals based on the Dutch educational system. For analyses, the Verhage levels were subsequently converted into approximate years of education in accordance with previous studies, allowing comparison across participants.^13^ The study was approved by the Medical Ethical Committee of Amsterdam University Medical Center. All participants provided written informed consent for the use of their medical data for research purposes. Consent was obtained according to the Declaration of Helsinki.

**RESEARCH IN CONTEXT**

1. **Systematic review**: A literature review was conducted on PubMed to identify studies that link cognitive screening outcomes to everyday functioning in Alzheimer's disease (AD). However, few relevant studies were identified. Relevant citations are provided.
2. **Interpretation**: Our findings highlight that cognition and daily functioning are related but distinct constructs. Even individuals with higher cognitive scores experienced difficulties in specific daily activities, such as working, using a computer, and managing the household budget. Among participants with the lowest cognitive scores, filling in forms, managing the household budget, and using a computer were most affected.
3. **Future directions**: Combining a global cognitive screener with functional assessment, such as the Amsterdam Instrumental Activities of Daily Living Questionnaire (A‐IADL‐Q‐30), is important for disease monitoring in clinical practice and trials. Future research should focus on longitudinal models that predict changes in daily functioning based on cognitive performance, to improve understanding of disease progression and guide personalized treatment decisions.

### 2.2 Amyloid measurement

Amyloid positivity was determined using cerebrospinal fluid (CSF) biomarkers. For CSF analysis, amyloid positivity was evaluated based on Aβ1‐42 concentrations. Until 2018, sandwich enzyme‐linked immunosorbent assays (ELISAs) were utilized (Innotest, Fujirebio, Gent, Belgium), with Aβ1‐42 values adjusted using drift correction.^14^ Since 2018, concentrations in CSF have been analyzed with Elecsys (Roche, Rotkreuz, Switzerland). For Innotest assays, a drift‐corrected Aβ1‐42 value below 813 pg/mL was considered positive, whereas for Elecsys assays, local cutoffs of <1000 pg/mL indicated positive amyloid status.^14^, ^15^

### 2.3 Measures

#### 2.3.1 Global cognitive screeners

Global cognitive functioning was assessed using the MMSE^5^ and the MoCA,^16^ both using a 0–30 score range, with higher scores indicating better cognitive performance. The MMSE is a cognitive screening tool that evaluates orientation, memory recall, object naming, attention, and visuoconstruction. Psychometrically, the MMSE demonstrated high internal consistency and moderate to high test–retest reliability (ranging from 0.60 to 0.95).^17^ It has shown discriminatory value in differentiating dementia from healthy aging.^18^, ^19^, ^20^ The MoCA was developed to detect MCI and other forms of cognitive decline, assessing executive function, memory processes, and attention.^16^ It has demonstrated high test–retest reliability (0.86–0.92)^16^, ^21^ and high sensitivity in detecting MCI (90%) and mild AD (100%).^16^ Compared to the MMSE, the MoCA showed higher diagnostic accuracy in distinguishing between normal cognition and cognitive decline.^22^, ^23^ Previous studies have demonstrated that demographic factors, such as age, education level, IQ, and socioeconomic status, can influence performance on both cognitive screening instruments.^24^, ^25^, ^26^

#### 2.3.2 Everyday functioning

The A‐IADL‐Q‐30 was developed to evaluate early functional (IADL) impairments, specifically in individuals with MCI and dementia.^27^

Each item was scored on a Likert scale ranging from 0 (no difficulty performing an activity) to 4 (unable to perform an activity due to difficulties with their memory, planning, or thinking), with intermediate values representing increasing levels of difficulty: 1 (slightly more difficulty), 2 (more difficult), and 3 (much more difficult). Current performance was then compared with scores from the 4 weeks preceding the assessment. The A‐IADL‐Q‐30 total score is computed using item response theory (IRT) modeling, in which observed item responses are linked to an underlying, latent construct.^28^, ^29^ The resulting IRT‐derived scores are expressed as *T*‐scores, which are standardized with a mean of 50 and an SD of 10 in a memory clinic population. Lower *T*‐scores indicate greater functional impairment.^30^ The A‐IADL‐Q‐30 is completed by a caregiver or an informant who knows the patient well, such as a relative, friend, spouse, or partner. When the study partner did not know whether the activity was performed or if the reason for not performing an activity was not related to cognitive problems, the item was considered missing and was not included in the analysis. The A‐IADL‐Q‐30 has been validated extensively,^27^ with robust psychometric properties, including high internal consistency and test‐retest reliability,^31^ good construct validity,^32^ adequate diagnostic accuracy,^33^ good responsiveness to change,^34^, ^35^ good cross‐cultural validity,^36^ clinical meaningfulness,^30^ and available normative data.^37^

### 2.4 Statistical analyses

All analyses were performed in R version 4.3.2,^38^ using the “Stats” package. Differences between diagnostic groups in age, education in years, and A‐IADL‐Q‐30, MMSE, and MoCA were tested using analysis of variance (ANOVA), and the Tukey Honest Significant Difference (HSD) test was applied to investigate the underlying pairwise group differences. Gender differences were evaluated using chi‐square tests for categorical data. Separate multiple regression analyses were performed with MMSE and MoCA test scores as independent variables, and the A‐IADL‐Q‐30 as dependent variable, adjusted for age, gender, and years of education. These analyses were performed in the total sample and in the diagnostic groups separately.

In addition to the main analyses, we conducted exploratory analyses to visualize the associations and aid clinicians in interpretation. For this purpose, we divided the cognitive scores into quartiles along with the corresponding A‐IADL‐Q responses. Within each quartile, we explored the proportion of reported problems using cutoffs that have been established to facilitate the interpretation of the A‐IADL‐Q‐30.^30^ Score categories were defined as follows: no problems (*T*‐score ≥60), mild problems (*T*‐score 50–59), moderate problems (*T*‐score 40–49), and severe problems (*T*‐score ≤40). Chi‐square test was used to compare A‐IADL‐Q‐30 problems across the MMSE and MoCA quartiles, to determine if they align. Due to a discrepancy between the number of completed MMSE (*n *= 1,228) and MoCA (*n *= 678) assessments, we performed a sensitivity analysis in which MoCA scores were converted to MMSE equivalents using a crosswalk.^39^ This crosswalk allows scores obtained with one test to be used to infer equivalent scores on the other test, facilitating comparability across participants. Finally, we described the most commonly affected A‐IADL‐Q‐30 activities at the item level across the quartiles, calculating percentages based on the valid responses. Analyses were conducted both at the level of impairment categories (no, mild, moderate, severe) and at the item level. For these item‐level analyses, missing data were not imputed. After this step we dichotomized the response categories into “no problems” and “any problems” to enhance interpretability. Given the exploratory nature of the analyses, no formal pairwise testing or correction for multiple comparisons was performed.

## 3 RESULTS

Demographic and clinical characteristics of the participants are shown in Table 1. On average, participants were 64.7 ± 7.6 years (mean ± SD) years old, and 627 were female (51%). The median years of education was 10 years, with an interquartile range (IQR) of 4 years. Diagnostic groups differed in terms of age (with MCI participants being the oldest; 66.2 ± 6.6), education (with SCD and MCI participants receiving the most education; median education 13), and sex distribution (with dementia participants being predominantly female; *n *= 466, 55%; see Table 1).

**TABLE 1:** Demographic and clinical characteristics.

	Total	SCD	MCI	Dementia	*p*‐value
**N**	1.228	156	228	844	
**Age in years**	64.7 (7.6)	63.4 (8.4)	66.2 (6.6)	64.5 (7.7)	<0.001^b^
**Female, *n* (%)**	627 (51)	65 (42)	96 (42)	466 (55)	0.005^c^
**Education** ^a^ **, M (IQR)**	10 (5–17)	13 (6–17)	13 (5–17)	10 (5–17)	<0.001^b^
**A‐IADL‐Q‐30**					
*T*‐score^d^	50.6 (9.2)	58.3 (7.5)	55.6 (7.5)	47.7 (8.5)	<0.001^b^
	22.8–70.0	38.4–69.9	31.6–70.0	22.8–69.4	
MMSE	*n *= 1.228	*n *= 156	*n *= 228	*n *= 844	
	22.7 (5.4)	28.1 (1.5)	26.5 (2.3)	20.6 (5.2)	<0.001^b^
	2.0–30.0	22.0–30.0	17.0–30.0	2.0–30.0	
MoCA	*n *= 678	*n *= 97	*n *= 132	*n *= 449	
	19.4 (5.8)	25.3 (2.6)	23.3 (2.3)	17.0 (5.4)	<0.001^b^
	1.0–30.0	17.0–30.0	17.0–29.0	1.0–27.0	

*Note*: Results are presented as mean ± SD with the range, unless otherwise indicated. *p*‐values: comparison between diagnostic groups.

Abbreviations: A‐IADL‐Q‐30, Amsterdam Instrumental Activities of Daily Living Questionnaire; ANOVA, analysis of variance; IQR, Interquartile Range; MCI, mild cognitive impairment; MMSE, Mini‐Mental State Examination; MoCA, Montreal Cognitive Assessment; SCD, subjective cognitive decline.

^a^ Education: in years.

^b^ ANOVA.

^c^ Chi‐square test.

^d^ The score shown is based on either the original or short version of the A‐IADL‐Q.

### 3.1 Relationship between global cognition and daily functioning across clinical groups

**FIGURE 1:**
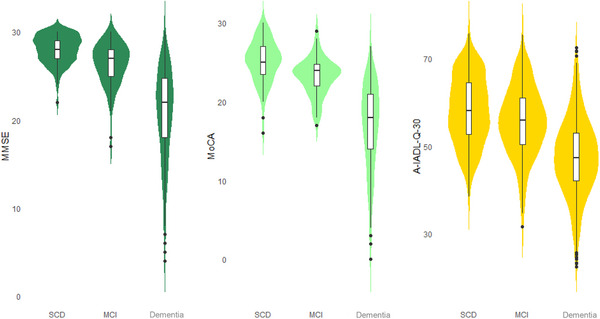
Distribution of MMSE, MoCA, and A‐IADL‐Q‐30 scores across diagnostic groups. *Note*: A‐IADL‐Q‐30 scores are presented as T‐scores. A‐IADL‐Q‐30, Amsterdam Instrumental Activities of Daily Living Questionnaire; MCI, mild cognitive impairment; MMSE, Mini‐Mental State Examination; MoCA, Montreal Cognitive Assessment; SCD, subjective cognitive decline.

As expected, global cognitive and functional performance was highest in the SCD group (A‐IADL‐Q‐30: 58 ± 8, MMSE: 28 ± 5, MoCA: 25 ± 3), intermediate in the MCI group (A‐IADL‐Q‐30: 56 ± 8, MMSE: 27 ± 2, MoCA: 23 ± 2), and lowest in the AD dementia group (A‐IADL‐Q‐30: 48 ± 9, MMSE: 21 ± 5, MoCA: 17 ± 5). All global cognitive and functional measures differed across diagnostic groups (see Table 1 and Figure 1).

### 3.2 The relationship between MMSE and MoCA scores and IADL performance

In our primary analyses, A‐IADL‐Q‐30 showed moderate correlations with the MMSE (*r* = 0.53, 95% confidence interval [CI] 0.48–0.57) and MoCA (*r* = 0.50, 95% CI 0.45–0.56). Multiple linear regression analyses, adjusted for age, sex, and education, showed that both MMSE (*B* = 0.96, 95% CI 0.87–1.04) and MoCA (*B* = 0.79, 95% CI 0.68–0.89) predicted A‐IADL‐Q‐30 scores, although the strength of these associations varied across diagnostic groups (Table 2). Despite these significant associations, the correlations were not strong enough to meet the assumptions required for direct linking methods.^10^

**TABLE 2:** Multiple regression analyses for MMSE and MoCA with A‐IADL‐Q‐30.

	Total		SCD		MCI		Dementia	
	*N* = 1.221		*n *= 155		*n *= 227		*n *= 839	
MMSE Model	*B*	*β*	*B*	*β*	*B*	*β*	*B*	*β*
	[95% CI]	[95% CI]	[95% CI]	[95% CI]	[95% CI]	[95% CI]	[95% CI]	[95% CI]
**Constant**	31.45^***^ [26.71, 36.19]		43.62^***^[19.39, 67.85]		39.26^***^[24.39, 54.13]		35.48^***^ [29.83, 41.12]	
**MMSE**	0.96^***^ [0.87, 1.04]	0.54^***^[0.49, 0.59]	0.54 [−0.30, 1.38]	0.11[−0.17, 0.78]	0.58^*^ [0.13, 1.03]	0.17^*^ [0.07, 0.58]	0.74^***^ [0.63, 0.85]	0.43^***^[0.35,0.48]
**Age**	−0.06 [−0.12, 0.00]	−0.05 [−0.10, 0.00]	−0.07 [−0.21, 0.08]	−0.07 [−0.17, 0.07]	−0.09 [−0.24, 0.07]	−0.07 [−0.19, 0.05]	−0.04 [−0.11, 0.03	−0.04 [−0.09, 0.03]
**Education** ^a^	−0.01 [−0.18, 0.15]	−0.00 [−0.05, 0.05]	0.26 [−0.17, 0.70]	0.10 [−0.05, 0.21]	0.25 [−0.10, 0.60]	0.10 [−0.03,0.18]	−0.16 [−0.36, 0.05]	−0.05 [−0.11, 0.01]
**Sex**	0.80 [−0.12, 1.70]	0.04 [−0.01, 0.09]	0.37 [−2.12, 2.86]	0.02 [−0.11, 0.15]	2.49^*^ [0.48, 4.50]	0.16^*^ [0.03, 0.27]	0.72 [−0.38, 1.82]	0.04 [−0.02, 0.10]

MoCA model	*N* = 678		*n *= 97		*n *= 132		*n *= 449	
**Constant**	36.36^***^[30.52, 42.20]		23.88^*^ [3.63, 44.13]		54.59^***^[36.52, 72.65]		37.79^***^[30.69, 44.88]	
**MoCA**	0.79^***^ [0.68, 0.89]	0.51^***^[0.41, 0.54]	1.20^***^ [0.62, 1.79]	0.41^***^ [0.37, 1.08]	0.25 [−0.30, 0.79]	0.08 [−0.18, 0.48]	0.58^***^[0.44, 0.72]	0.39^***^ [0.27, 0.43]
**Age**	−0.00 [−0.08, 0.07]	−0.00 [−0.06, 0.06]	0.03 [−0.14, 0.19]	0.03 [−0.11, 0.15]	−0.11 [−0.31, 0.10]	−0.09 [−0.25, 0.08]	0.02 [−0.07, 0.11]	0.02 [−0.06, 0.09]
**Education** ^a^	−0.04 [−0.25, .17]	−0.01 [−0.08, 0.05]	0.26 [−0.26, 0.78]	0.10 [−0.08, 24]	0.17 [−0.28, 0.62]	0.07 [−0.08, 0.19]	−0.11 [−0.38, 0.15]	−0.04 [−0.11, 0.05]
**Sex**	0.10 [−1.07, 1.27]	0.01 [−0.06, 0.07]	−1.07 [−4.15, 2.01]	−0.07 [−0.22, 0.11]	0.13 [−2.39, 2.65]	0.01 [−0.13, 0.14]	0.54 [−0.90, 1.98]	0.03 [−0.05, 0.10]

*Note*: In this analysis, the MMSE and MoCA act as independent variables predicting A‐IADL‐Q‐30 (dependent variable). *B* = unstandardized *b*‐values; *β*, standardized *b*‐values.

Abbreviations: CI, confidence interval; MCI, Mild Cognitive Impairment; MMSE, Mini‐Mental State Examination; MoCA, Montreal Cognitive Assessment; *n *, sample size, varies across different models due to unequal subsample; SCD, subjective cognitive decline.

^a^ Education in years.

* *p* < 0.05.

*** *p* < 0.001.

### 3.3 Exploratory descriptive analyses of reported functional (IADL) problems across MMSE quartiles

**FIGURE 2:**
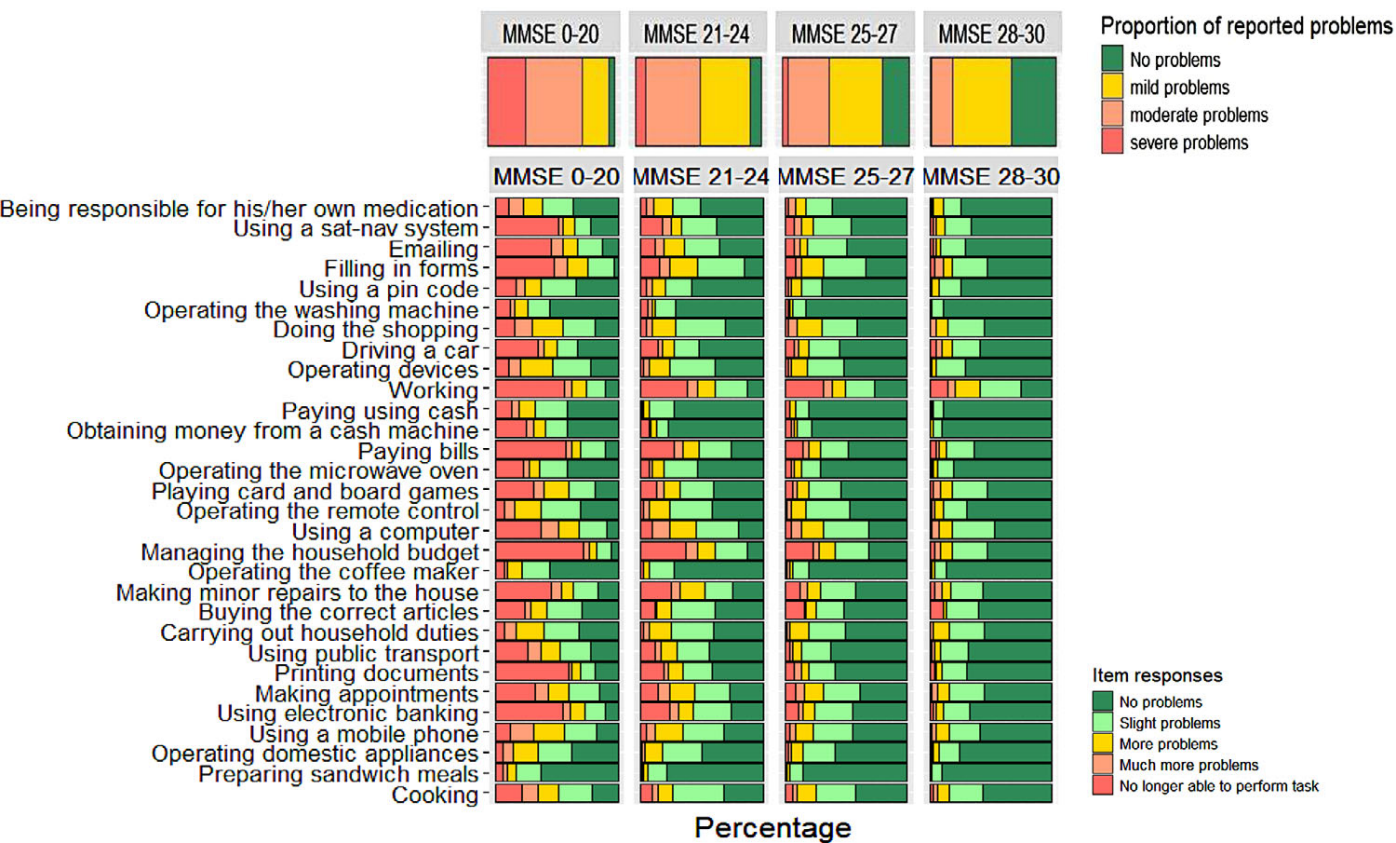
Proportion of reported problems and item responses across MMSE quartiles. *Note*: Proportion of reported problems: based on A‐IADL‐Q‐30 total score, categories shown for each MMSE quartile. Quartiles are based on total scores of the MMSE (*n *= 1228), ranging from 0 to 30. Item responses are based on the percentage of answers endorsed. MMSE, Mini‐Mental State Examination.

To provide insight into impairments in IADLs across levels of cognition, participants were divided into MMSE quartiles. Figure 2 illustrates the proportion of reported IADL problems across MMSE quartiles, including item‐level endorsement frequencies. Based on A‐IADL‐Q 30 cutoff scores, participants were further categorized into levels of functional (IADL) impairment.

A chi‐square test revealed significant differences in the proportion of A‐IADL‐Q problems between all MMSE quartiles *χ* ^2^ (3) = 116.91, *p* < 0.001. In the lowest MMSE quartile (0–20), functional difficulties were most impaired. Only 5% (*n *= 18) of participants in this group were classified as having “no problems” in daily functioning, whereas 21% (*n *= 78), 45% (*n *= 163), and 29% (*n *= 108) showed “mild,” “moderate,” and “severe problems,” respectively. The most frequently affected activities in this group were filling in forms (*n *= 260, 96%), managing the household budget (*n* = 169, 95%), and using a computer (*n *= 231, 90%).

In the second lowest quartile (21–24), 9% reported “no problems” (*n* = 28). The majority experienced “moderate problems” (*n *= 133, 43%), followed by “mild problems” (*n *= 125, 40%) and “severe problems” (*n *= 26, 8%). Working (*n *= 134, 88%) was the most frequently affected activity, followed by managing the household budget (*n *= 146, 87%), and filling in forms (*n *= 219, 86%). For participants in the third quartile (25–27), 21% reported “no problems” (*n *= 68) in daily functioning. “Mild problems” (*n *= 132, 42%) were most frequently reported, followed by “moderate problems” (*n *= 105, 33%), and 4% reported “severe problems” (*n *= 12). Difficulties were more frequently reported in working (*n *= 113, 74%), using a computer (*n *= 193, 69%), and managing the household budget (*n *= 119, 68%).

Among participants in the highest MMSE quartile (28–30), 35% (*n *= 82) reported no problems in daily functioning. The majority experienced “mild problems” (*n *= 108, 47%), whereas “moderate” (*n *= 39, 17%) and “severe problems” (*n *= 3, 1%) were observed less often. Within this group, the most commonly affected activities were working (*n *= 84, 74%), followed by using a computer (*n *= 112, 52%) and managing the household budget (*n *= 71, 47%). Across all MMSE quartiles, activities such as preparing sandwich meals, operating the coffeemaker, and obtaining money from a cash machine were generally preserved. For MoCA, we found comparable results, which are available in Figure S1. Sensitivity analyses using the crosswalk‐generated scores yielded largely similar results. Figure S2 shows the item‐level difficulty for the linked MoCA scores derived from the crosswalk.

**FIGURE 3:**
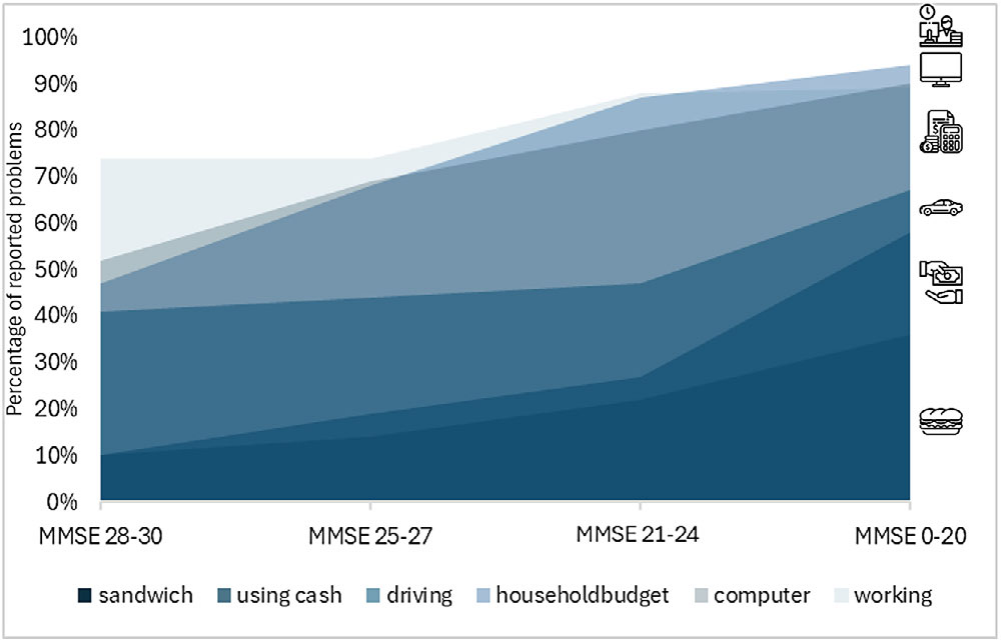
Reported problems in selected activities across MMSE quartiles. *Note*: Participants were categorized into quartiles based on their MMSE total scores (ranging 0–30), with higher scores on the left and lower scores on the right of the x‐axis. The y‐axis reflects reported problems in six daily activities, with a higher position indicating more reported problems. This figure presents cross‐sectional data and illustrates reported problems across levels of cognitive performance. MMSE, Mini‐Mental State Examination.

Figure 3 illustrates how problems in six specific activities vary across different levels of cognitive performance (MMSE score), based on cross‐sectional data. We selected the two least‐affected activities across quartiles, preparing sandwich meals and paying using cash, and the three most affected activities from the highest quartile, managing the household budget, using a computer, and working. In addition, driving was included because it is frequently reported as highly relevant by patients and caregivers. The two least‐affected activities (preparing sandwich meals and paying using cash) show a gradual increase in reported problems with decreased cognitive performance. In contrast, the three most affected activities (managing the household budget, using a computer, and working) already show difficulties at higher MMSE scores, with the percentage of problems increasing as cognitive impairment worsens. Problems with driving remain relatively stable in the first two MMSE quartiles, before increasing in the second lowest quartile, followed by further deterioration. For MoCA, we found comparable results, which are available in the Figure S3.

## 4 DISCUSSION

Our main finding is that, although a direct linkage could not be established, higher levels of cognitive functioning were moderately associated with a higher level of overall everyday functioning in amyloid‐positive individuals. Exploratory descriptive analysis indicated that in the highest (28–30) and second‐highest MMSE quartiles (25–27), the most affected activities were working, using a computer, and managing the household budget. In the second‐lowest quartile (21–24), difficulties in working, managing the household budget, and filling in forms were most reported. In the lowest MMSE quartile (0–20), the most affected activities were filling in forms, managing the household budget, and using a computer. Our cross‐sectional findings also seem to imply that some everyday activities decline gradually (e.g., preparing sandwich meals), whereas other activities (e.g., driving a car) decline more abruptly once cognitive performance reaches a certain level of impairment.

Previous research has shown varying results regarding the strength of the association between more traditional IADL measures and global cognition,^17^ with weaker associations in pre‐dementia stages compared to dementia. We explored whether, with this amyloid‐positive sample, a translation of performance on cognitive screeners to IADL functioning would be possible. We found that the relationship between the two, while showing a large effect size in the overall sample, was too low for direct statistical linking.^10^, ^40^ This is also reflected in our finding that, within groups, individuals with similar global cognitive performance experienced varying levels of difficulty with everyday activities, both on the total score and specific activities. An interesting observation is that even in the group diagnosed with dementia, a small minority of participants (*n = *65, 8%) reported no functional (IADL) difficulties on the A‐IADL‐Q‐30, suggesting that diagnosis may also rely on a broader clinical evaluation.

Potential explanations include the influence of factors on everyday functioning beyond cognition, such as physical, social, and environmental factors.^41^, ^42^ Still, the A‐IADL‐Q‐30 was previously found to be less prone to contextual factors compared to other conventional IADL instruments.^27^, ^43^ It is therefore also likely that, despite being related to everyday functioning, commonly used cognitive screeners are insufficiently able to fully capture the complexity of these everyday activities. However, we provide insight by demonstrating differences in IADL performances between the quartiles, as these indicate that IADLs change alongside cognitive decline.

In addition, the strength of associations between the A‐IADL‐Q‐30 and cognitive screeners varied across the diagnostic groups: the MoCA was most strongly associated in the SCD group, whereas the MMSE showed stronger associations in the dementia group. This likely reflects differences in sensitivity, with the MoCA capturing early cognitive variability through executive function measures, and the MMSE being limited by a known ceiling effect in higher‐functioning individuals.^40^ These results highlight that the relationships between functional performance and cognitive scores differ depending on the assessment tool and diagnostic group.

Previously it was demonstrated that IADL functioning declines over different cognitive stages in amyloid‐positive individuals.^44^ In the present cross‐sectional study, we identified which activities are most affected across the different cognitive quartiles. Our findings are consistent with previous studies into specific daily activities. For example, a longitudinal study reported that amyloid was significantly associated with decline in financial capacity in cognitively normal participants as well as those with MCI.^45^ Similar decline in moderate AD was also previously described.^46^ In our study, almost half of the participants with the highest cognitive functioning level also reported difficulties in managing the household budget and paying with cash in the lower levels of cognitive functioning. Another activity that participants across the different quartiles often reported as difficult was using a computer. Consistent with our findings, previous research has shown that the use of computer applications, such as word processing, email, and internet browsing, was associated with overall cognitive functioning.^47^ Although computer use persists among individuals with MCI, it shows both quantitative declines (less time, fewer sessions) and qualitative changes (use of different or less complex applications).^47^ Participants in the lowest cognitive quartiles reported more driving difficulties. Longitudinal studies show that individuals with very mild to mild dementia often continue driving for a long time despite these challenges.^48^ Driving relies on multiple cognitive processes—attention, memory, decision‐making—that may be unevenly affected.^49^ A recent study reported that lower performance in memory, visual attention, mental flexibility, and visuoconstruction was associated with more difficulties in daily functioning.^50^ Cognitive screeners such as the MMSE do not fully capture deficits in these domains, limiting their ability to predict challenges in complex activities such as driving.

Our findings support the use of both functional (IADL) measures and cognitive screening tools to improve the prediction of future functioning in clinical practice and research. Previous research has shown that combining cognitive and functional measures improves sensitivity to detecting disease progression, as demonstrated by instruments such as the Cognitive‐Functional Composite (CFC) in early stages of AD.^34^ These findings may support the development or refinement of both cognitive and IADL measures, which could contribute to more accurate tracking of subtle changes in cognitive and functional (IADL) abilities, especially in early stages of the disease. Such refined measures would not only improve monitoring of disease progression but also enhance the evaluation of treatment effects, facilitating early interventions and more personalized treatment approaches.

The strengths of this study include the large sample size of 1228 amyloid‐positive participants across different stages of global cognitive performance, enhancing the generalizability and robustness of our findings. Our sample included individuals with MCI and mild dementia who could potentially be eligible for DMTs, allowing us to examine how cognitive status, as measured by commonly used cognitive screeners, relates to everyday functioning in this clinically relevant population. Another strength is the use of a widely validated functional assessment tool, with the A‐IADL‐Q‐30 specifically shown to be highly sensitive to early functional impairments in AD.^27^ In addition, this study included a broad range of complex everyday activities across various categories, such as household tasks, leisure activities, and the use of appliances.

Several limitations must be taken into account when interpreting these results. First, the cross‐sectional design restricts insight into how daily functioning and specific activities evolve over time. Longitudinal data‐driven approaches are needed to clarify activity‐specific trajectories and their relationship with global cognitive performance. In addition, unmeasured factors such as physical functioning, depressive symptoms, and comorbidities may also influence functional (IADL) decline and could help clarify how global cognitive performance relates to daily functioning. Third, no formal correction for multiple comparisons was applied to the exploratory analyses, such as the quartile‐based comparisons. These analyses provide valuable insights but should be interpreted with appropriate caution. Finally, the group of participants with complete MoCA was smaller than the group with complete MMSE, which could affect the validity of the comparison between the two screening tools. However, a sensitivity analysis in which MoCA scores were converted to MMSE equivalents yielded largely similar results, suggesting that this discrepancy did not substantially influence the findings.

Previous research shows that patients and their care partners often have a need for information regarding their future functioning, including both cognitive and functional performance.^6^, ^7^ Daily functioning is an important aspect of life for many patients and their care partners, which is often reflected in their concerns about performing activities such as driving a car or pursuing hobbies.^6^, ^7^ Recent predictive models for MCI and dementia, involving the MMSE, meet the need for information regarding patients' future cognitive trajectories.^7^ Our findings add that by combining a global cognitive screener with a functional assessment, such as the A‐IADL‐Q‐30, we provide insights into how global cognitive performance relates to problems an individual may experience in daily functioning. In addition, focusing on specific activities could provide a clearer picture of disease progression and the impact of new DMTs, potentially facilitating better communication with patients and care partners.

Future research should aim to predict the timing and progression of specific functional (IADL) limitations in relation to cognitive decline. This approach emphasizes the importance of understanding when certain impairments emerge. Our findings underscore the necessity of collecting longitudinal data on both cognitive performance and everyday functioning to develop predictive models. Such models could improve our understanding of individual disease trajectories and enhance decision‐making regarding prognosis and treatment.

In conclusion, this cross‐sectional study offers valuable insights into the relationship between performance‐based global cognitive screening tools and everyday functioning in amyloid‐positive individuals in a memory clinic setting. Even individuals with the highest cognitive functioning faced difficulties in specific daily tasks, underscoring the complexity of real‐world functioning. Recognizing the specific daily activities most affected by cognitive decline at various cognitive stages is essential for evaluating clinical meaningfulness of treatment effects, personalized care, and future research.

## CONFLICT OF INTEREST STATEMENT

E.G., C.T., A.H., W.F., and S.S. are recipients of TAP‐dementia (www.tap‐dementia.nl), receiving funding from ZonMw (#10510032120003) in the context of Onderzoeksprogramma Dementie, part of the Dutch National Dementia Strategy. TAP‐dementia receives co‐financing from Avid Radiopharmaceuticals and Amprion. E.G. has received research support from NWO, ZonMw, Hersenstichting, and Health∼Holland. E.G. has performed contract research for Heuron Inc., Roche, and 1st Biotherapeutics. E.G. has a consultancy agreement with IXICO and Life Molecular Imaging for the reading of PET scans. Research of C.T. is supported by the European Commission (Marie Curie International Training Network [MIRIADE grant number 860197 and TAME grant number 101119596]; Innovative Medicines Initiatives 3TR [Horizon 2020 grant number 831434], EPND [IMI 2 Joint Undertaking grant number 101034344], and JPND [bPRIDE, CCAD]; and the European Partnership on Metrology, co‐financed from the European Union's Horizon Europe Research and Innovation Programme and by the Participating States [22HLT07 NEuroBioStand; Horizon Europe PREDICTFTD 101156175]), the CANTATE project (funded by the Alzheimer's Drug Discovery Foundation), Alzheimer's Association, Michael J Fox Foundation, Health Holland, the Dutch Research Council (ZonMW), Alzheimer's Drug Discovery Foundation, and The Selfridges Group Foundation, Alzheimer Netherlands. C.T. is recipient of ABOARD, which is a public‐private partnership receiving funding from ZonMW (#73305095007) and Health∼Holland, Topsector Life Sciences & Health (PPP‐allowance; #LSHM20106). C.T. has research contracts with Acumen, ADx Neurosciences, AC‐Immune, Alamar, Aribio, Axon Neurosciences, Beckman‐Coulter, BioConnect, Bioorchestra, Brainstorm Therapeutics, C2N diagnostics, Celgene, Cognition Therapeutics, EIP Pharma, Eisai, Eli Lilly, Fujirebio, Instant Nano Biosensors, Merck, Muna, Novo Nordisk, Olink, PeopleBio, Quanterix, Roche, Toyama, Vaccinex, and Vivoryon. C.T. is Editor in Chief of *Alzheimer's Research and Therapy*, and serves on editorial boards of *Molecular Neurodegeneration*, *Alzheimer's & Dementia*, *Neurology: Neuroimmunology & Neuroinflammation*, and *Medidact Neurologie/Springer*, and is committee member to define guidelines for Cognitive disturbances, and one for acute Neurology in The Netherlands. C.T. has consultancy/speaker contracts for Aribio, Biogen, Beckman‐Coulter, Cognition Therapeutics, Danaher, Eisai, Eli Lilly, Janssen, Merck, Novo Nordisk, Novartis, Olink, Roche, Sanofi, and Veravas. A.H. was advisor to the Brain Research Center and Eli Lilly. Research programs of A.H. have been funded by ZonMW and Alzheimer Nederland (WE.06‐2021‐06). All funding is paid to her institution. W.F. is a recipient of the IHI‐ PROMINENT project (grant agreement No. 101112145). W.F. and S.S. are recipients of IHI‐AD‐RIDDLE project (grant agreement No. 101132933). PROMINENT and AD‐RIDDLE are supported by the Innovative Health Initiative Joint Undertaking (IHI JU). The JU receives support from the European Union's Horizon Europe research and innovation programme and COCIR, EFPIA, EuropaBio, MedTech Europe and Vaccines Europe, with Davos Alzheimer's Collaborative, Combinostics OY., Cambridge Cognition Ltd., C2N Diagnostics LLC, and neotiv GmbH. W.F. is recipient of ABOARD, which is a public–private partnership receiving funding from ZonMW (#73305095007) and Health∼Holland, Topsector Life Sciences & Health (PPP‐allowance; #LSHM20106). Research programs of Wiesje van der Flier have been funded by ZonMW, NWO, EU‐JPND, EU‐IHI, Alzheimer Nederland, Hersenstichting CardioVascular Onderzoek Nederland, Health∼Holland, Topsector Life Sciences & Health, stichting Dioraphte, Noaber foundation, Pieter Houbolt Fonds, Gieskes‐Strijbis fonds, stichting Equilibrio, Edwin Bouw fonds, Pasman stichting, Philips, Biogen MA Inc, Novartis‐NL, Life‐MI, AVID, Roche BV, Eli‐Lilly‐NL, Fujifilm, Eisai, and Combinostics. W.F. holds the Pasman chair. W.F. has been an invited speaker at Biogen MA Inc, Danone, Eisai, WebMD Neurology (Medscape), NovoNordisk, Springer Healthcare, and the European Brain Council. All funding is paid to her institution. W.F. is consultant to Oxford Health Policy Forum CIC, Roche, Biogen MA Inc, Eisai, Eli‐Lilly, and Owkin France. W.F. participated in advisory boards of Biogen MA Inc, Roche, and Eli Lilly. W.F. is member of the steering committee of phase 3 EVOKE/EVOKE+ studies (NovoNordisk). W.F. is a member of the steering committee op Phase 3 Trontinemab study (Roche); all funding is paid to her institution. W.F. is a member of the steering committee of PAVE and Think Brain Health. W.F. is a member of the Scientific Leadership Group of InRAD. W.F. was associate editor of *Alzheimer's, Research & Therapy* in 2020/2021. W.F. is associate editor at *Brain*. W.F. is member of Supervisory Board (Raad van Toezicht) Trimbos Institut. S.S. is a recipient of funds from Health∼Holland, Topsector Life Sciences & Health (PPP allowance: DEFEAT‐AD, LSHM20084; Remote‐DEM, LSHM22026), Alzheimer Nederland (SPREAD+ # WE.32‐2022‐01), and Ministry of Health, Welfare and Sports (#90001586), ZonMw in the context of Onderzoeksprogramma Dementie, part of the Dutch National Dementia Strategy (TAP‐dementia, #10510032120003), ZonMW (VIMP, #7330502051 and #73305095008, NWO (YOD‐MOLECULAR, #KICH1.GZ02.20.004) as part of the NWO Research Program KIC 2020‐2023 MISSION—Living with dementia. YOD‐MOLECULAR receives co‐financing from Winterlight Labs, ALLEO Labs, and Hersenstichting. Team Alzheimer also contributes to YOD‐MOLECULAR. The remaining authors declare that the research was conducted in the absence of any commercial or financial relationships that could be construed as a potential conflict of interest. Any author disclosures are available in the Supporting Information.

## CONSENT STATEMENT

All participants provided informed consent.

## Supporting information



Supporting information

Supporting information

Supporting information

Supporting information
