# Supplementary material for: The relationship between cognitive screeners and everyday functioning in amyloid‐positive participants from the Amsterdam Dementia Cohort
Source: Alzheimers Dement (Amst). 2026 Jan 4;18(1):e70233. doi: 10.1002/dad2.70233 (PMC12765400; doi:10.1002/dad2.70233)
Supplement: Supplementary file 2 — Supporting information [file DAD2-18-e70233-s003.pdf]

Supplementary figure 1: Proportion of reported problems and item responses across MoCA quartiles.

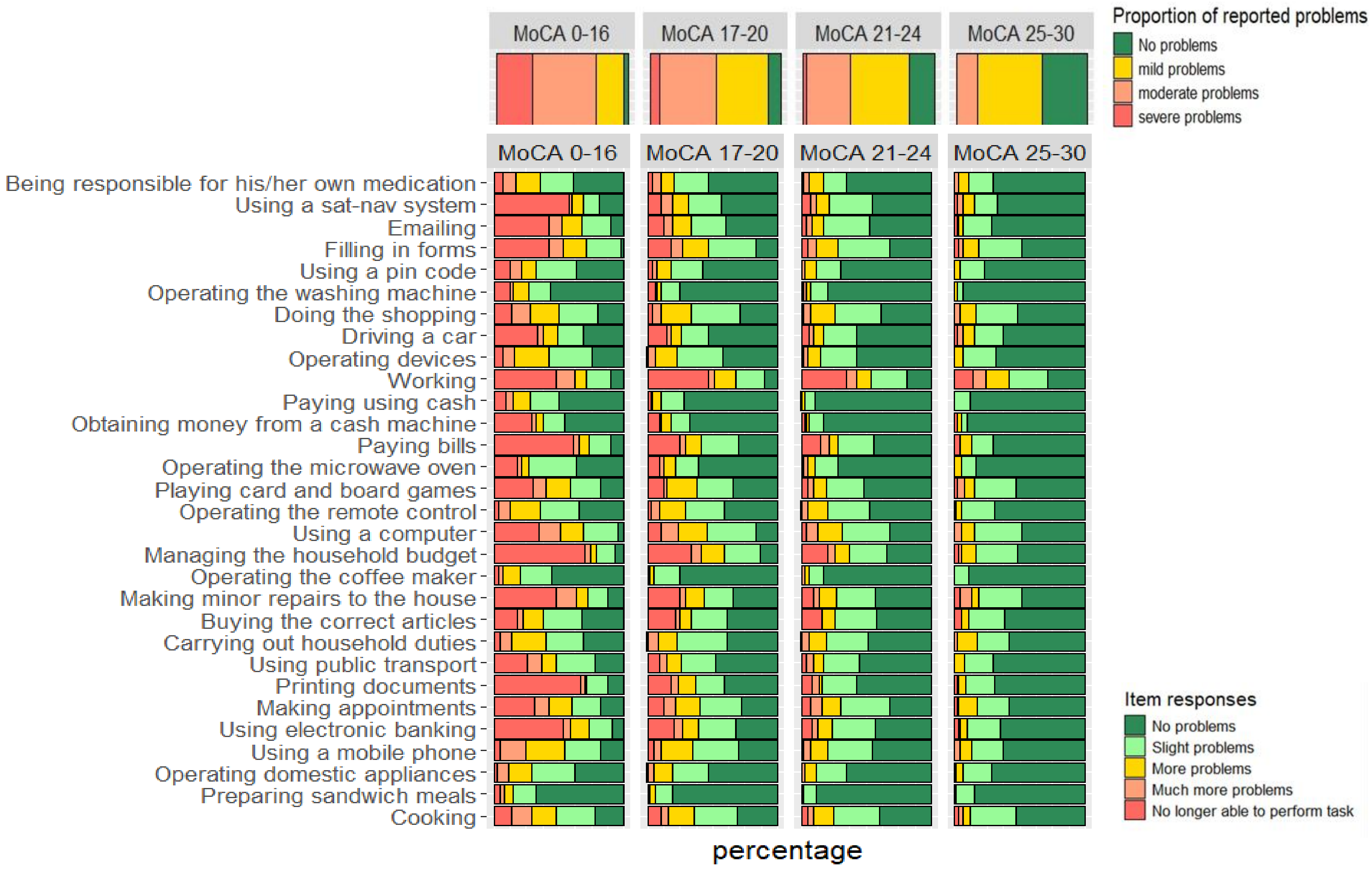


NOTE. Proportion of reported problems: based on A-IADL-Q total score categories shown for each MoCA quartile. Quartiles are based on total scores of the MoCA (n=678), ranging from 0 to 30. Item responses based on percentages of answers endorsement. Abbreviations: MoCA: Montreal Cognitive Assessment
