# Supplementary material for: The relationship between cognitive screeners and everyday functioning in amyloid‐positive participants from the Amsterdam Dementia Cohort
Source: Alzheimers Dement (Amst). 2026 Jan 4;18(1):e70233. doi: 10.1002/dad2.70233 (PMC12765400; doi:10.1002/dad2.70233)
Supplement: Supplementary file 3 — Supporting information [file DAD2-18-e70233-s001.pdf]

Supplementary figure 2: Proportion of Reported Problems and Item Responses Across Linked MoCA Scores (Cross-walk).

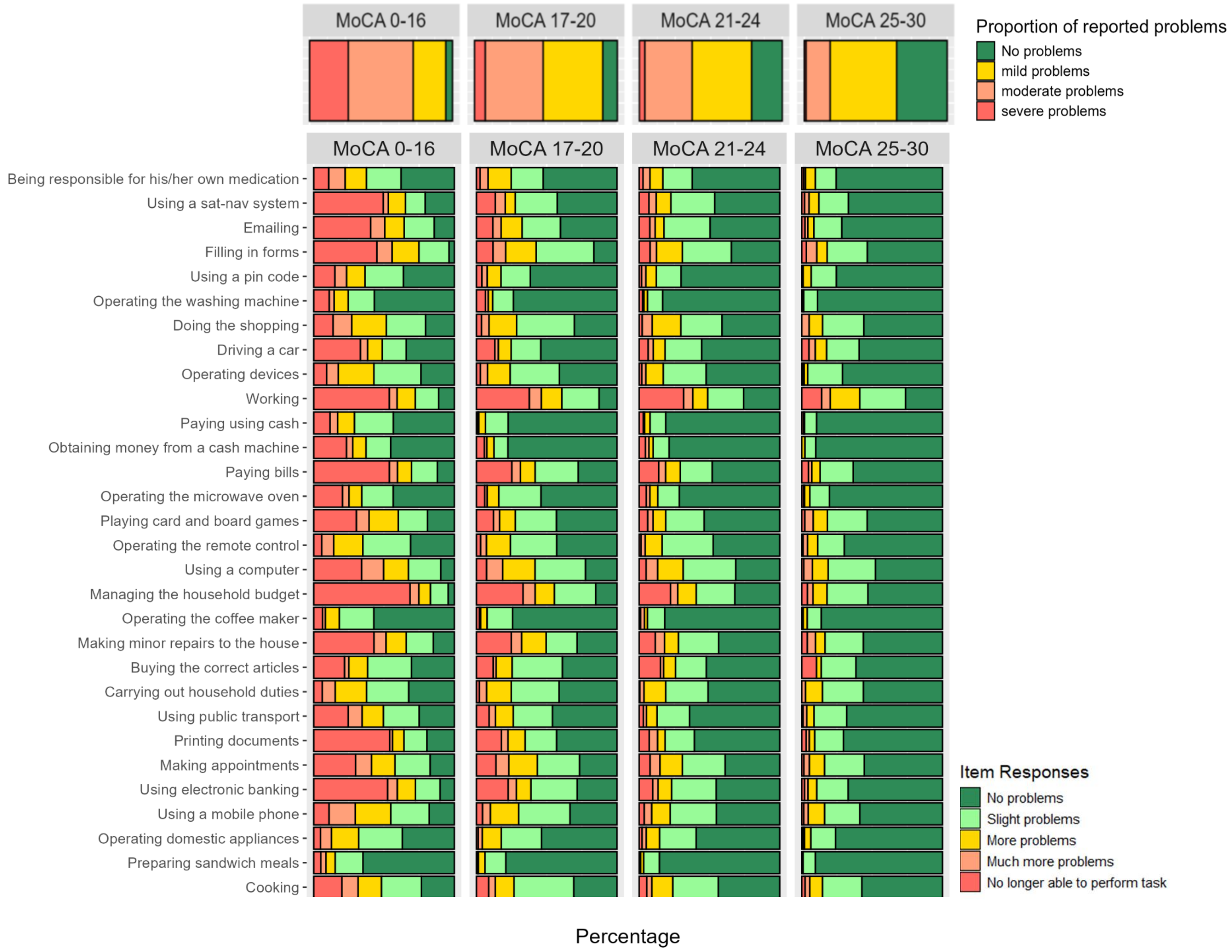

NOTE. Proportions of reported problems were based on A-IADL-Q total score categories. These categories are shown for each MoCA crosswalk score group. MoCa scores were derived from MMSE Groups (N=1,228) reflect total scores from the Moca crosswalk (range 0-30). Item responses are presented as percentages of answer endorsement. Abbreviations: MoCA: Montreal Cognitive Assessment
