# Supplementary material for: The relationship between cognitive screeners and everyday functioning in amyloid‐positive participants from the Amsterdam Dementia Cohort
Source: Alzheimers Dement (Amst). 2026 Jan 4;18(1):e70233. doi: 10.1002/dad2.70233 (PMC12765400; doi:10.1002/dad2.70233)
Supplement: Supplementary file 4 — Supporting information [file DAD2-18-e70233-s004.pdf]

Supplementary figure 3: Reported problems in selected activities across MoCA quartiles.

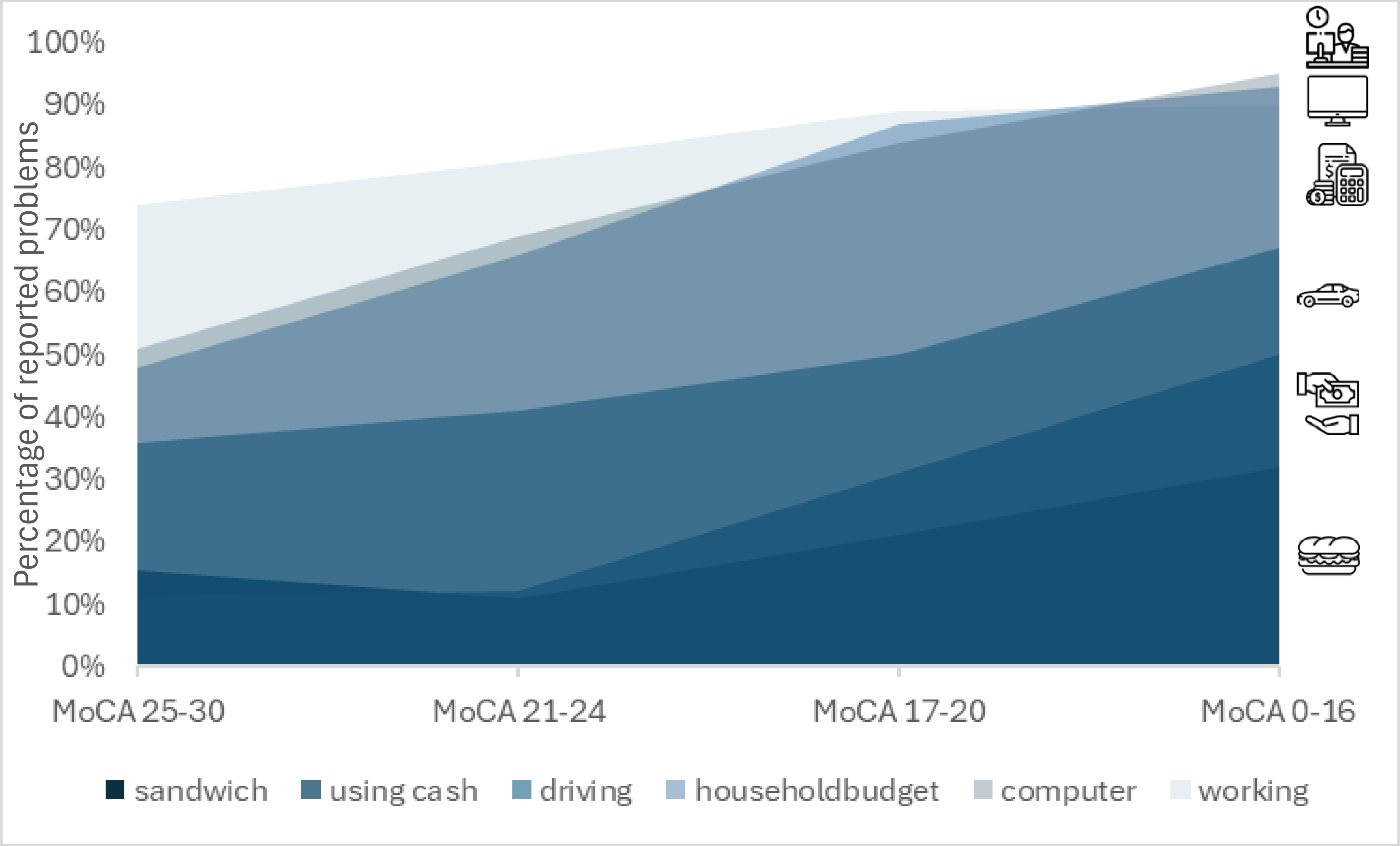

NOTE. Participants were categorized into quartiles based on their MoCA total scores (ranging 0-30), with higher scores on the left and lower scores on the right of the x-axis. The y-axis reflects reported problems in six daily activities, with higher position indicating more reported problems. This figure presents cross-sectional data and illustrates reported problems across levels of cognitive performance. Abbreviations: MoCA: Montreal Cognitive Assessment
